# Photocatalytic Oxygen Evolution under Visible Light Mediated by Molecular Heterostructures

**DOI:** 10.3390/molecules28227500

**Published:** 2023-11-09

**Authors:** Zhaoqi Shen, Yujie Zhang, Guang Zhang, Shiyong Liu

**Affiliations:** 1 School of Metallurgy and Chemical Engineering, Jiangxi University of Science and Technology, Ganzhou 341000, China; 18857337582@163.com (Z.S.); 15079698527@163.com (Y.Z.); 2 Department of Chemistry, Tianjin University, Tianjin 300072, China

**Keywords:** perylene diimide, molecular heterostructures, oxygen evolution rate

## Abstract

Due to their structural and property tunability, semiconductive conjugated polymers (CPs) have emerged as promising candidates for photocatalytic water splitting. Compared with inorganic materials, the photocatalytic performance of mono-component polymers was limited by the fast recombination of photoexcited charge carriers, and they always needed to catch up to expectations. To this end, researchers established molecular donor–acceptor heterostructures, which could notably promote oxygen production efficiency due to their more effective charge carrier separation. In this work, easy Schiff base reactions between side-chain -CHO groups and terminal -NH_2_ groups were used to introduce benzene and perylene diimide (PDI) into the molecular heterostructure to serve as electron donors (D) and electron acceptors (A). In particular, for the first time, we employed the molecular heterostructures of CPs to promote photocatalytic O_2_ production. One prepared molecular heterostructure was demonstrated to improve oxygen generation rate (up to 0.53 mmol g^−1^ h^−1^) through visible light-driven water splitting. Interestingly, based on the photoelectric properties, a stepwise two-electron/two-electron pathway constituted the photocatalytic mechanism for oxygen production with the molecular heterostructure. These results provide insights into designing and fabricating high-performance molecular heterostructures for photocatalytic oxygen production.

## 1. Introduction

The solid worldwide energy dependence on non-renewable fossil fuels has triggered severe social and environmental problems, such as the energy crisis and global warming [1,2,3,4,5,6]. Motivated by natural photosynthesis, artificial photocatalytic systems provide promising routes to resolve energy-related problems by harnessing sunlight [7,8,9,10]. In 1980, inorganic materials (TiO_2_ electrodes) were, for the first time, reported to mediate the photoelectrochemical-induced water splitting into hydrogen and oxygen [11]. Until now, inorganic materials have been the most widely studied photocatalysts because of their high stabilities and charge carrier mobilities [12]. Compared with inorganic materials, conjugated polymers exhibit their specific advantages as another kind of photocatalyst [13,14,15,16,17,18]. For example, polymers are mainly composed of naturally abundant lightweight elements (i.e., C, N, O, and S, etc.), and both the architectures and properties of polymers could be easily tailored by delicate structure design [19]. However, most mono-component polymers suffer from low solar-to-oxygen conversion efficiencies due to the fast recombination of photogenerated charge carriers [20,21]. Thus, exploiting the diversity of polymer structures to develop novel CPs with efficient photocatalytic properties is extremely important [10].

For inorganic materials, the heterojunction composed of two kinds of semiconductors with staggered absorption bands was demonstrated to expand the light absorption range and enhance the charge carrier mobility and separation efficiency, which, therefore, could further improve the conversion efficiency of solar energy [22,23,24,25]. As such, similarly, polymer heterojunctions [26,27,28,29,30] were constructed to optimize light absorption and enhance exciton dissociation. For example, Shen et al. [31] developed three polymer heterojunctions composed of fluorene-based polymers and g-C_3_N_4_, which achieved an AQY of 27% under 440 nm of light irradiation. Li et al. first constructed molecular heterostructures based on covalent triazine frameworks (CTFs) via a sequential polymerization strategy to produce H_2_. Molecular heterostructures of electron-accepting benzothiadiazole and electron-donating thiophene moieties showed much better photocatalytic properties than single-component polymers [32]. Photophysical and electrochemical characterizations indicated that, compared to mono-component polymers, the efficient charge separation with the molecular heterostructures can be more readily fabricated in principle by incorporating donor and acceptor units into different polymers’ skeletons [32].

Herein, we developed a new polymer-based molecular heterostructure and explored its structure–property relationship associated with light-induced water splitting into oxygen. As reported in [33], Zhu et al. disclosed that the crystalline perylene diamide (PDI)-functionalized polymer (Urea-PDI) mediated active oxygen production without cocatalysts, with a high oxygen evolution rate (3223.9 μmol h^−1^ g^−1^) which was over 107.5 times as high as those of PDI-based supramolecular photocatalysts under visible light. We first synthesized PDI-based mono-component conjugated polymers (BPDI) via imide condensation [34] between p-phenylenediamine and 3,4,9,10-perylenetetracarboxylic acid anhydride. BPDI exhibited subtle photoactivity for O_2_ production in water/DMF solution under visible light irradiation due to the deficient thermodynamic driving force. Then, 2,5-Dibromoterephthalaldehyde with 3,4-ethoxylene dioxy thiophene, benzene, and dibenzothiophene sulfone groups used to prepare the precursors was named BEDOT, BB, and BSO, respectively. Interestingly, three corresponding molecular heterostructures formed when three linear polymers (BEDOT, BB, and BSO) were covalently linked with BPDI by an aldimine condensation reaction. The enhanced photocatalytic properties of the targeted molecular heterostructures were attributed to the intrinsic merits of the molecular heterostructures, e.g., light absorption, photoluminescence (PL), transient photocurrent response, and charge carrier mobilities. Via delicate design of the precursors, the staggered band alignments of the molecular heterostructure could optimize the energy gaps of polymers to provide both broad photoresponse and enough driving force for light-induced water splitting. On the other hand, the abundant inter- and intramolecular donor-acceptor (D–A) interactions and highly conjugated backbones of the molecular heterostructures could effectively enhance charge carrier mobility and separation efficiency, which were pivotal for enhancing photocatalytic performance. The prepared heterostructure BB/BPDI exhibited remarkable oxygen production rates of 0.53 mmol h^−1^ g^−1^ under visible light irradiation. This work demonstrates that molecular heterostructure is an efficient strategy to improve the photoactivities of conjugated polymers and provides insights into the design of high-performance photocatalysts in different fields.

## 2. Results and Discussions

### 2.1. Synthesis and Characterizations

**Scheme 1:**
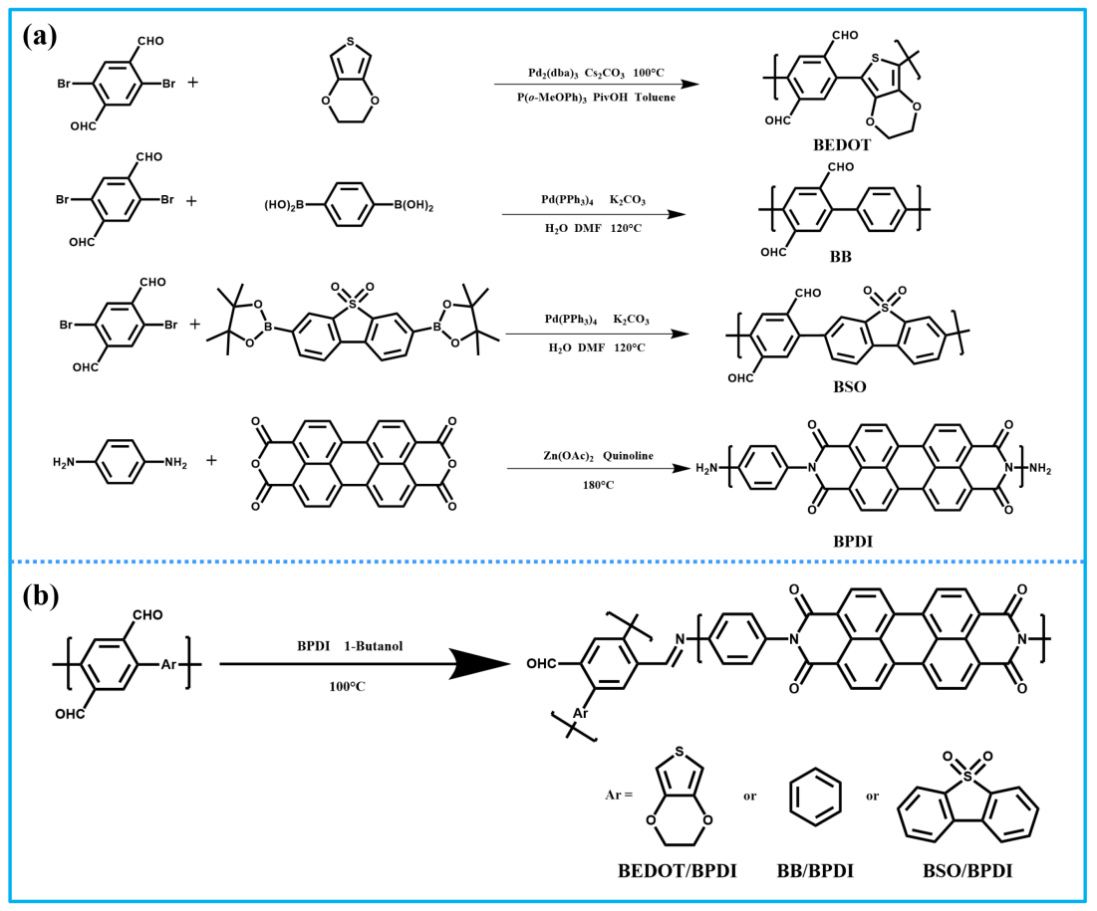
Synthetic routes of (**a**) pristine polymers and (**b**) their corresponding heterostructures.

According to reported procedures, both linear conjugated polymers (BSO and BB) were synthesized via Suzuki–Miyaura coupling between 2,5-dibromoterephthalaldehyde and diboronic acid of the aromatic cores ((5,5-dioxidodibenzo[b,d]thiophene-3,7-diyl)diboronic acid and 1,4-phenylenediboronic acid). BEDOT was prepared via Pd-catalyzed direct C-H arylation polymerization (DArP) between 3,4-ethoxylene dioxy thiophene (EDOT) and 2,5-dibromoterephthalaldehyde, with an equivalent feed ratio. For synthesizing molecular heterostructures, the BPDI was incorporated into BEDOT, BB, and BSO via Schiff base reaction (Scheme 1). Here, PDI and dibenzo[b,d]thiophene-5,5-dioxide were employed as electron-withdrawing units, while EDOT and benzene were used as substantial electron donors and weak electron donors, respectively. All polymers precipitated during the reaction were insoluble in common organic solvents, e.g., MeOH, toluene, tetrahydrofuran, CH_2_Cl_2_, and CHCl_3_.

**Figure 1:**
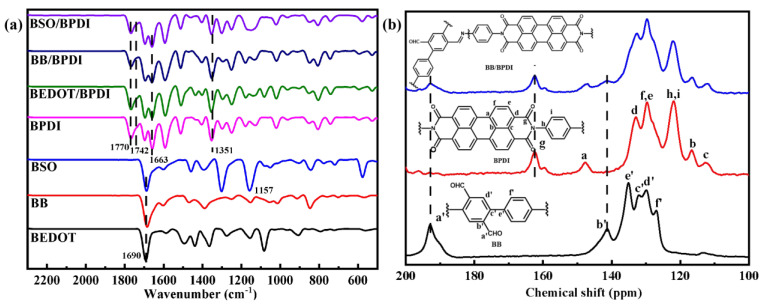
(**a**) FT-IR spectrum of all polymers; (**b**) solid state ^13^C cross-polarization magic-angle-spinning nuclear magnetic resonance spectra of BB, BPDI, and BB/BPDI.

The Fourier transform infrared (FT-IR) spectra (Figure 1a) of the resultant CPs revealed evident signals in the spectrum. The two prominent characteristic bands observed at 1770 and 1742 cm^−1^ were attributed to the carbonyl group’s asymmetric and symmetric stretching, respectively. The -CHO groups of the three precursors (BB, BSO, and BEDOT) were observed at 1690 cm^−1^. Specifically, for BPDI, the characteristic band belonging to amic acid intermediate product was observed at around 1663 cm^−1^, which also could be found in the other three molecular heterostructures [35]. The FT-IR spectra (Figure S1) of monomers and polymers provides additional evidence. Meanwhile, the band around 1351 cm^−1^ was assigned to the -C-N-C- in the PDI units. In addition, for BSO and BSO/BPDI, the characteristic signals at around 1157 cm^−1^ belong to the O=S=O group, which is not found in others [36]. Interestingly, these corresponding signals were also exhibited in the structure curve of molecular heterostructures and were consistent with our expectations, confirming the proposed structures. Solid state ^13^C cross-polarization magic-angle-spinning nuclear magnetic resonance spectra (Figure 1b) of polymers presented a broad peak from 110 ppm to 150 ppm, which was assignable to the signals of aromatic carbons [37]. The characteristic C=O signals from PDI units in BPDI and BB/BPDI were located at 162 ppm, and the ^13^C signal of aldehyde groups appeared at around 192 ppm. Their co-presence in the molecular heterostructure of BB/BPDI indicated the successful introduction of both functional segments in the heterostructure skeleton. Moreover, the results of transmission electron microscopy (TEM) and scanning electron microscopy (SEM) (Figures S2 and S3) showed that the morphologies of the linear BEDOT, BB, and BSO polymers exhibited similar stacked sheets, which indicates that the three linear polymers have a similar structure arrangement at the microscale level. Furthermore, BPDI exhibited a distinct morphology that appeared as aggregated nanoparticles. Interestingly, all of these molecular heterostructures demonstrated a composite appearance, encompassing stacked sheet and aggregated nanoparticle structures. This consistent feature also indicates that the molecular heterostructure could be prepared by the method of this study.

### 2.2. Photophysical and Electrochemical Properties

**Figure 2:**
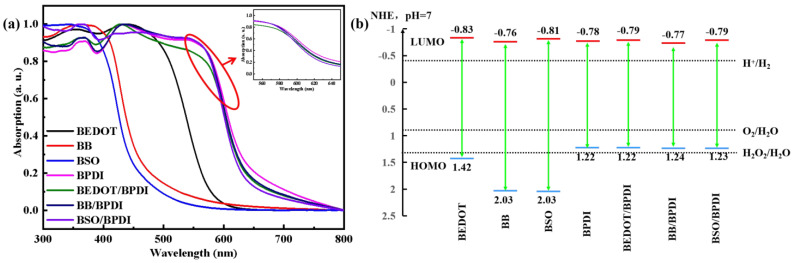
(**a**) Normalized UV-vis adsorption spectra of the prepared CPs (inset: UV-vis adsorption spectra of BPDI, BEDOT/BPDI, BB/BPDI, and BSO/BPDI from 550 to 650 nm.); (**b**) HOMO (HOMO = LUMO − E_g_) and LUMO (LUMO determined from cyclic voltammetry) levels of the prepared CPs.

We also characterized the optical properties and analyzed the electrochemical properties of mono-component linear polymers and the molecular heterostructures. The UV-vis diffuse reflection spectroscopy (Figure 2a) of BEDOT, BB, and BSO exhibited absorption edges at 500 nm, 540 nm, and 600 nm, respectively, whereas that of BPDI and molecular heterostructures had broader light adsorption up to ~700 nm. Compared with the pristine (BEDOT, BB, and BSO) polymers, all of the molecular heterostructures exhibited broader absorption. The optical bandgap (E_g_) was calculated according to the absorption onsets of UV-vis absorption by plotting (*αhν*)^2^ against *hν* based on the Kubelka–Munk function. Cyclic voltammetry (CV, Figure S4a) was carried out to provide the lowest unoccupied molecular orbital (LUMO) levels, and the highest occupied molecular orbital (HOMO) levels were calculated from the difference between the E_g_ and the LUMO level. As shown in Figure 2b, BEDOT, BB, and BSO manifested appropriate HOMOs to offer sufficient thermodynamic driving forces to mediate a light-driven oxygen evolution half-reaction. The HOMO level of BPDI can only produce oxygen via a four-electron pathway rather than the stepwise two-electron/two-electron pathway [10]. The reduction in the bandgap of the polymer is a well-known prerequisite for achieving a broader light absorption region. However, to drive efficient photocatalytic reactions, it is crucial to have adequate thermodynamic driving forces. With the charge transfer between the donor unit (benzene) and the acceptor unit (PDI), this staggered band alignment enabled the BB/BPDI to effectively absorb visible light and offer sufficient thermodynamic driving forces from the LUMO of BB.

**Figure 3:**
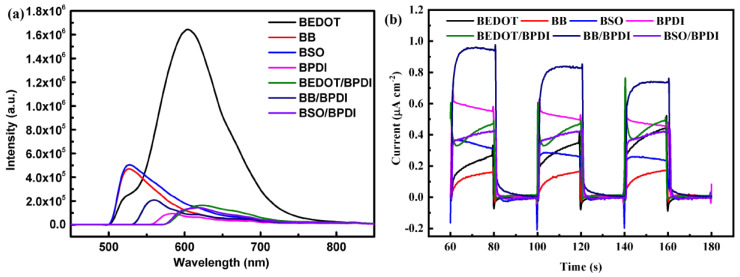
(**a**) Steady-state fluorescence spectra of the prepared CPs. (**b**) Transient photocurrent of the prepared CPs under full-arc light.

Steady-state photoluminescence (PL) spectroscopy (Figure 3a) was used to gain insight into the capabilities of all polymers and probe their internal electron transfer dynamics. The light emission spectra of BEDOT, BB, and BSO exhibited a strong peak at 604 nm, 531 nm, and 530 nm, respectively, due to the fast recombination of the photogenerated electron and hole. In contrast, the PL intensities of the molecular heterostructures were quenched, and those molecular heterostructures exhibited similar PL intensities. The attenuated PL intensities of molecular heterostructures compared with mono-component linear polymers reflected that the photogenerated charge recombination in the molecular heterostructures was notably charge-suppressed [38]. Compared with mono-component linear polymers with D-A effects, molecular heterostructures with intermolecular D-A effects were designed to establish enhanced charge separation performance, which was attributed to the covalent bridge limiting the recombination of excitons between the donor units and acceptor units [32].

In addition, photocurrent measurements (Figure 3b) and electrochemical impedance spectra (Figure S4b) were performed. As shown in Figure 3b, the molecular heterostructures possessed much higher photocurrent densities than the linear CPs (BEDOT, BB, and BSO). In addition, the charge carrier density of BEDOT/BPDI was estimated to be higher than that of BSO/BPDI, which was attributed to the electron push–pull effect between the donor and acceptor along the conjugate skeleton. BB/BPDI exhibited the highest current intensity at 0.84 μA cm^−2^. These results indicate that the molecular heterostructures with intermolecular D-A effects could effectively improve photogenerated charge carrier transfer. Electrochemical impedance spectroscopy (EIS) also evidenced that BB/BPDI had a more negligible interfacial charge transfer resistance than BSO/BPDI and BEDOT/BPDI. The above results confirm that the efficient charge carrier transfer across the covalently limited molecular heterostructure was one of the decisive keys for the tremendous photocatalytic performance.

### 2.3. Photocatalytic Performances

**Figure 4:**
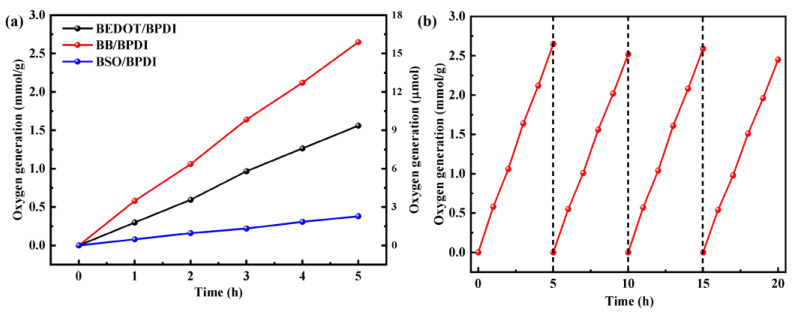
(**a**) Oxygen evolution as a factor of time mediated by molecular heterostructures. (**b**) Cycling tests of O_2_ evolution for BB/BPDI (evaluated every 5 h).

The photocatalytic O_2_ evolution capabilities of the as-prepared polymers were evaluated using AgNO_3_ as an electron scavenger and La_2_O_3_ as a pH buffer agent under visible light irradiation. Approximately 2 wt% of cobalt cocatalyst was photodeposited in situ on the surfaces of the photocatalysts via a facile blending strategy. The control experiment was without any photocatalysts and did not produce any oxygen, and the other control experiments were also shown in Table 1. The physical mixture of BB and BPDI, as depicted in Table 1, exhibited the subtly oxygen production rate under visible light. This phenomenon highlights the crucial role of molecular heterostructures with covalent bridges in promoting visible light-induced oxygen generation. In addition, by increasing or decreasing the BPDI ratio x in BB/BPDI to synthesize BB/BPDI-2 (x = 2) and BB/BPDI-0.5 (x = 0.5), both B-B/BPDI-2 and B-B/BPDI-0.5 showed prominent activity decreases under similar conditions. Unfortunately, the oxygen evolution rates of BPDI, BEDOT, BB, and BSO were not found, which could be attributed to the rapid charge recombination or insufficient thermodynamic driving force. In particular, the rare photocatalytic activity of BPDI indicated that the mechanism for photocatalytic water splitting into oxygen was a stepwise two-electron/two-electron pathway [10]. Figure 4a presents the time-dependent hydrogen evolution curves, displaying a linear increase versus time for BEDOT/BPDI, BB/BPDI, and BSO/BPDI over five hours. Additionally, the normalized efficiencies for light-induced water splitting into oxygen, calculated by normalizing the photocatalyst weights, are listed in Figure 4a (left Y axis). The molecular heterostructures BEDOT/BPDI and BSO/BPDI exhibited average photocatalytic activities of 0.31 and 0.08 mmol g^−1^ h^−1^, respectively. The correctly hybridized BB/BPDI exhibited an improved photocatalytic activity, with a maximum oxygen production value of 0.53 mmol g^−1^ h^−1^ due to the excellent charge mobility and charge separation efficiency. It agrees with our preliminary optical and electrochemical results for the molecular heterostructures.

The recycling stability of BB/BPDI was evaluated in the paper. A continuous photocatalytic reaction was conducted for 20 h, with intermittent evacuation every 5 h under visible light irradiation. To ensure the consistency of the photocatalyst system, the exact amounts of AgNO_3_ and La_2_O_3_ were added to the reaction mixture every cycle. As shown in Figure 4b, the photocatalytic system maintains its continuous stability for each photocatalytic cycle. Even after 20 h, only a marginal decline was observed in the oxygen evolution rate (OER) during the four consecutive cycles. One interesting observation was that after the photocatalyst reaction, the black particles appeared at the bottom of the reactor after 10 min, which could be attributed to the sacrificial agent AgNO_3_ being reduced to form Ag nanoparticles during oxygen production. While the phenomenon might have affected the absorption of visible light by the photocatalyst, it did not significantly impact its stability or efficiency.

**Figure 5:**
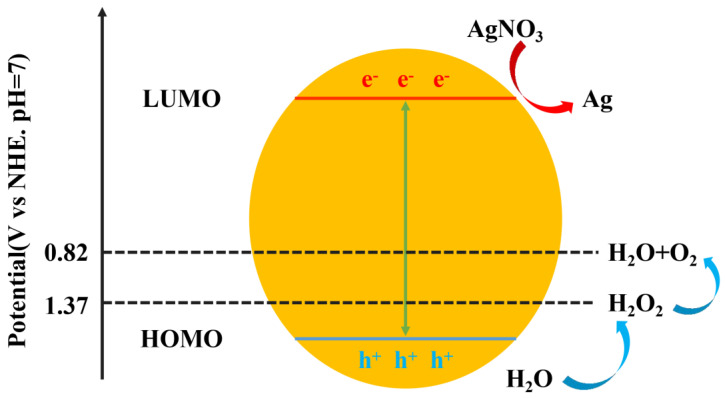
OER pathways on BB/BPDI.

Based on the previous reports [11,39,40], a photocatalytic mechanism with the stepwise two-electron/two-electron pathway is proposed for the molecular heterostructures (Figure 5). When the photon energy equals the bandgap of the polymer photocatalyst, photoinduced e^−^-h^+^ pairs are produced and separated. Then, the photogenerated electrons and holes from electron donors and electron acceptors can be promptly recombined via the charge transfer along the covalent bridge. In contrast, the remaining photogenerated holes and electrons retain separated counterparts to drive water-splitting reactions. Meanwhile, the covalent bridge of molecular heterostructures is a medium in which charge is carried along by the polymer skeleton, which also can limit the recombination of charge in the electron donor. The remaining holes and electrons further diffuse to the surface and migrate to the active sites, i.e., the photocatalyst/water interface. The photogenerated h^+^ is captured by adsorbed H_2_O to generate · OH and H^+^ at the active sites. The hydroxyl radicals play a crucial role in further forming the primary intermediate (H_2_O_2_) to promote the photocatalytic water splitting. The generated H_2_O_2_ dissociates into H_2_O and O_2_ via the simultaneous two-electron redox reaction. Co-species as metal co-catalysts can effectively appeal charge transfer to the sacrificial agent (AgNO_3_), promoting photocatalytic water splitting to generate O_2_ rather than H_2_ exclusively. Then, AgNO_3_ sequentially consumes the generated electrons to produce the Ag particles, limiting intramolecular and intermolecular charge recombination.

## 3. Materials and Methods

### 3.1. Chemicals and Materials

2,5-dibromoterephthalaldehyde, Benzene-1,4-diboronic acid, 3,4-ethylenedioxythiophene, p-Phenylenediamine, 3,4,9,10-Perylenetetracarboxylic Dianhydride (PDI), 3,7-Dibromodibenzothiophene-5,5-Dioxide, Bis(pinacolato)diboron, Tetrahydroxydiboron, 1,1′-Bis(diphenylphosphino)ferrocene-palladium(II)dichloride dichloromethane complex, Pd(PPh_3_)_4_, Pd_2_(dba)_3_ (98%), Zn(OAc)_2_ (99%), PivOH (99%), KOAc (99.9%), K_2_CO_3_ (99.9%), Cs_2_CO_3_ (99.9%), P(o-MeOPh)_3_ (98%), and anhydrous 1,4-Dioxane were purchased from commercial sources and used without further purification. Anhydrous toluene was obtained via distillation following treatment with calcium hydride.

### 3.2. Synthesis of All Polymers

Synthesis of 3,7-bis(4,4,5,5-tetramethyl-1,3,2-dioxaborolan-2-yl)dibenzo[b,d]thiophene 5,5-dioxide was conducted as follows: 3,7-Dibromodibenzothiophene-5,5-Dioxide (2.673 mmol, 1000 mg), Bis(pinacolato)diboron (5.868 mmol, 1490 mg), 1,1′-Bis(diphenylphosphino)ferrocene-palladium(II)dichloride dichloromethane complex (0.160 mol, 131 mg), and KOAc (1570 mg) were added into a Schlenk tube and sealed under vacuum. A total of 40 mL anhydrous 1,4-Dioxane was added to the tube and degassed three times via the freeze–pump–thaw cycle. Then, the tube was rigorously stirred at 100 °C for 32 h under an argon atmosphere. After cooling to room temperature, the undissolved crude product was purified by soxhlet extraction using CH_2_Cl_2_, methanol, and water to obtain the solid powder. Finally, the product was dried in a vacuum for 10 h at 60 °C and powders of polymer BPDI were obtained, respectively.

Synthesis of BPDI was conducted via the synthetic procedures as follows. p-Phenylenediamine (60.64 mg, 1.1 eq), 3,4,9,10-Perylenetetracarboxylic Dianhydride (200 mg, 0.5102 mmol, 1 eq), and Zn(OAc)_2_ (40 mg) were added into a Schlenk tube and sealed under vacuum. A total of 8 mL quinoline was added to the tube and degassed three times via the freeze–pump–thaw cycle. Then, the tube was rigorously stirred at 120 °C for 72 h under an argon atmosphere. After cooling to room temperature, the undissolved crude product was purified by soxhlet extraction using CH_2_Cl_2_, methanol, and water to obtain the solid powder. Finally, the product was dried in a vacuum for 10 h at 60 °C and powders of polymer BPDI were obtained respectively.

Synthesis of BSO and BB was conducted as follows: diboronic acid of the aromatic cores (3,7-bis(4,4,5,5-tetramethyl-1,3,2-dioxaborolan-2-yl)dibenzo[b,d]thiophene 5,5-dioxide, 1 eq, 0.685 mmol, 320.7 mg; Benzene-1,4-diboronic acid, 1 eq, 0.685 mmol, 113.5 mg), 2,5-dibromoterephthalaldehyde (1 eq, 200 mg), K_2_CO_3_ (3 eq, 283.6 mg), and Pd(PPh_3_)_4_ (0.05 eq, 39.6 mg) were added into a Schlenk tube and sealed under vacuum. The solvent was added to the tube (10 mL DMF and 1 mL H_2_O) and degassed three times via the freeze–pump–thaw cycle. Then, the tube was rigorously stirred at 120 °C for 72 h under an argon atmosphere. After cooling to room temperature, the undissolved crude product was purified by soxhlet extraction using CH_2_Cl_2_, methanol, and water to obtain the solid powder. Finally, the product was dried in a vacuum for 10 h at 60 °C and powders of polymer BSO and BB were obtained.

Synthesis of BEDOT was conducted as follows [41,42,43]: 3,4-ethylenedioxythiophene (1 eq, 0.685 mmol, 97.4 mg), 2,5-dibromoterephthalaldehyde (1 eq, 200 mg), Pd_2_(dba)_3_ (0.02 eq, 12.5 mg), Cs_2_CO_3_ (2 eq, 446.5 mg), P(o-MeOPh)_3_ (0.04 eq, 9.7 mg), and PivOH (0.3 eq, 21.0 mg) were added into a Schlenk tube and sealed under vacuum. A total of 10 mL anhydrous toluene was added to the tube and degassed three times via the freeze–pump–thaw cycle. Then, the tube was rigorously stirred at 110 °C for 48 h under an argon atmosphere. After cooling to room temperature, the undissolved crude product was purified by soxhlet extraction using CH_2_Cl_2_, methanol, and water to obtain the solid powder. Finally, the product was dried in a vacuum for 10 h at 60 °C and powders of polymer BEDOT were obtained.

Synthesis of BEDOT/BPDI, BSO/BPDI, and BB/BPDI was conducted via the synthetic procedures as follows. BPDI (100 mg, 1 eq) and another precursor (BEDOT, 1 eq, 60 mg; BB, 1 eq, 46.7 mg; BSO, 1 eq, 75.4 mg) were added to the tube and sealed under vacuum. A total of 5 mL 1-Butanol was added to the tube under an argon atmosphere and then rigorously stirred at 100 °C for 24 h. After cooling to room temperature, the undissolved crude product was purified by soxhlet extraction using CH_2_Cl_2_, methanol, and water to obtain the solid powder. The product was dried in a vacuum for 10 h at 60 °C and powders of polymer BEDOT/BPDI, BB/BPDI, and BSO/BPDI were obtained.

### 3.3. Characterization

All CPs were degassed at 60 °C for 12 h under vacuum (10–5 bar) before analysis. Unless otherwise specified, solid state ^13^C cross-polarization magic-angle-spinning nuclear magnetic resonance spectra were carried out on a Bruker Avance 400 model 400 MHz NMR spectrometer at a MAS rate of 10 kHz or were recorded on a Bruker AV200 spectrometer at 200 MHz and 50 MHz at room temperature. Infrared spectral measurement was carried out by making polymers with a KBr sheet on an FT-IR spectrometer (Bruker, ALPHA, Billerica, MA, USA) in transmission mode at room temperature. Morphologies of the polymers were obtained using a field emission scanning electron microscope (SEM, MLA650F, Rock Hill, SC, USA) and transmission electron microscope (TEM, Tecnai G2-20, Peabody, MA, USA).

The UV-vis diffuse reflection spectrum was obtained by means of a scanning UV-vis spectrophotometer (UV-2600, SHIMADZU, Kyoto, Japan) and BaSO_4_ as a reflectance sample. The photoluminescence of the polymers was measured with a fluorescence spectrometer (FL-1000, HORIBA, Shanghai, NJ, USA), with an excitation of 450 nm at room temperature. The photocurrents and cyclic voltammetry (CV) measurements were recorded using an electrochemical workstation (CHI650E/700E, CH Instruments Ins., Shanghai, China) in a conventional three-electrode cell. In this setup, a saturated calomel electrode (SCE) served as the reference electrode, while an Ag/AgCl electrode functioned as the counter electrode. The electrodes for photocurrents and CV measurements were immersed in a sodium sulfate electrolyte solution (0.1 M) and tetrabutylammonium hexafluorophosphate in acetonitrile (0.1 M), respectively. To simulate realistic photocatalytic reaction conditions, we employed a light source from Beijing Perfect Light, Beijing, China (PLS-SXE300) to induce the photocurrents of the photocatalyst. The measurement was conducted with a scan rate of 100 mV s^−1^ within the range of −1.6 to 1.6 V.

### 3.4. Measurement of Photocatalytic Activity

The hydrogen evolution experiments were performed on a LabSolar-III AG reaction cell (Beijing et al.). Typically, 6 mg of the photocatalyst was suspended in 30 mL H_2_O, 6 mL DMF, 25.6 mg AgNO_3_, and 31.5 mg La_2_O_3_, and two wt% Co(NO_3_)_2_ aqueous solution was added to this solution. The suspension was degassed to remove air and filled with N_2_. During the photocatalytic reaction, the polymers were irradiated by visible light (Beijing Perfect Light, Beijing, China, PLS-SXE300) from the light source (300 W Xe lamp). All of the gas was detected via online gas chromatography (GC9790, Zhejiang Fuli Analytical Instruments Corp., Wenling, China) at room temperature.

## 4. Conclusions

In conclusion, several molecular heterostructures composed of conjugated polymers were successfully prepared via the Schiff base reaction. The resultant BB/BPDI exhibited a much improved charge carrier separation efficiency, owing to its intermolecular D-A effects and the covalent bridge across the heterojunction. Most impressively, BB/BPDI mediated to have a remarkable oxygen production rate of 0.53 mmol g^−1^ h^−1^ under visible light irradiation, which was approximately higher than those of BEDOT/BPDI and BSO/BPDI. No oxygen was detected when the bare single-component linear conjugated polymers (BPDI, BB, BEDOT, and BSO) were applied. The molecular heterostructures with covalent bridges and intramolecular D-A interactions could improve the charge carrier transfer and separation. Based on the staggered bandgap alignment of the band segment, the stepwise two-electron/two-electron pathway was proposed to explain the photocatalytic mechanism. Our study provides insight into the materials’ rational design for efficient solar energy conversion. Additionally, the structural diversity of molecular heterostructures offers enormous potential for other photocatalytic fields, such as photocatalytic hydrogen evolution, organic transformations, or CO_2_ reductions.

## Figures and Tables

**Table 1:** Oxygen evolution rates (OER) of all polymers and corresponding blank control group.

Polymer	pH Buffer Agent	Sacrificial Agent	Co-Catalyst	Solvent	OER/mmol g^−1^ h^−1^
BEDOT	La_2_O_3_	AgNO_3_	Co(NO_3_)_2_	H_2_O/DMF	0
BB	La_2_O_3_	AgNO_3_	Co(NO_3_)_2_	H_2_O/DMF	0
BSO	La_2_O_3_	AgNO_3_	Co(NO_3_)_2_	H_2_O/DMF	0
BPDI	La_2_O_3_	AgNO_3_	Co(NO_3_)_2_	H_2_O/DMF	0
BEDOT/BPDI	La_2_O_3_	AgNO_3_	Co(NO_3_)_2_	H_2_O/DMF	0.31
BB/BPDI	La_2_O_3_	AgNO_3_	Co(NO_3_)_2_	H_2_O/DMF	0.53
BSO/BPDI	La_2_O_3_	AgNO_3_	Co(NO_3_)_2_	H_2_O/DMF	0.08
BB/BPDI	-	AgNO_3_	Co(NO_3_)_2_	H_2_O/DMF	0
BB/BPDI	La_2_O_3_	-	Co(NO_3_)_2_	H_2_O/DMF	0
BB/BPDI	La_2_O_3_	AgNO_3_	-	H_2_O/DMF	0
BB/BPDI	La_2_O_3_	AgNO_3_	Co(NO_3_)_2_	H_2_O	0
BB and BPDI	La_2_O_3_	AgNO_3_	Co(NO_3_)_2_	H_2_O/DMF	0

## Supplementary Materials

The following supporting information can be downloaded at: https://www.mdpi.com/article/10.3390/molecules28227500/s1, Figure S1: FT-IR spectrum of monomers, BB, BPDI and BB/BPDI. Figure S2: SEM images of the BPDI (a), BB (b), and BB/BPDI (c); TEM images of the BPDI (d), BB (e), and BB/BPDI (f). Figure S3: SEM images of the BEDOT (a), BEDOT/BPDI (b), BSO (c), BSO/BPDI (d). Figure S4: (a) CV curve of BSO, BB, BEDOT, and BPDI; (b) The electrochemical impedance spectra of BSO, BB, BEDOT, BPDI, BSO/BPDI, BB/BPDI, and BEDOT/BPDI.
