# Supplementary material for: Photocatalytic Oxygen Evolution under Visible Light Mediated by Molecular Heterostructures
Source: Molecules. 2023 Nov 9;28(22):7500. doi: 10.3390/molecules28227500 (PMC10673551; doi:10.3390/molecules28227500)
Supplement: Supplementary file 1 [file molecules-28-07500-s001.zip › molecules-2661593-supplementary.pdf]

Supporting Information

Photocatalytic Oxygen Evolution Under Visible Light Mediated by Molecular Heterostructures

Zhaoqi Shen[1], Yujie Zhang[1], Guang Zhang[2,*] and Shiyong Liu[1,*]

[1] School of Metallurgy and Chemical Engineering, Jiangxi University of Science and Technology, Ganzhou 341000, China;

[2] Department of Chemistry, Tianjin University, Tianjin 300072, China;

* Correspondence: chelsy@jxust.edu.cn (S.-Y. Liu); gzhdream@live.com (G. Zhang).

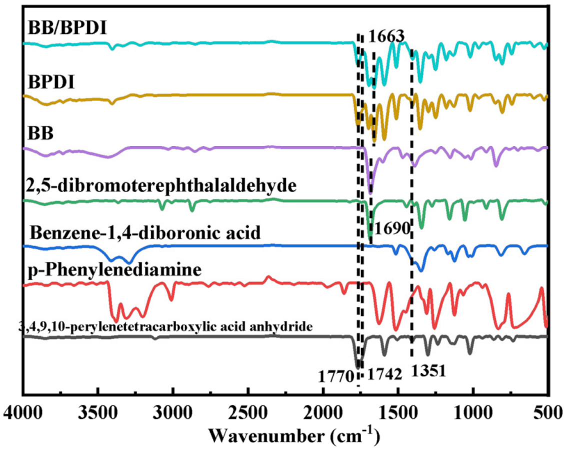

Figure S1. FT-IR spectrum of monomers, BB, BPDI and BB/BPDI.

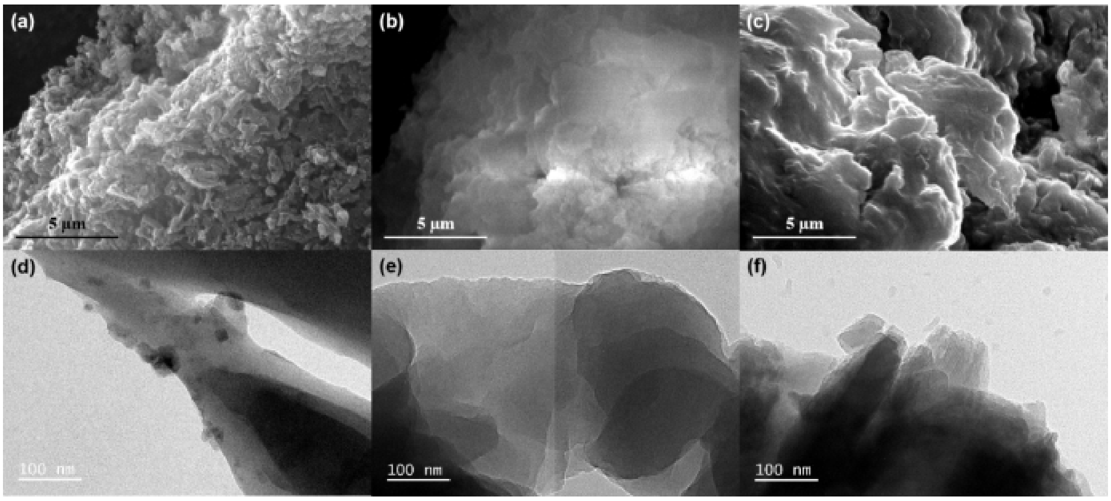

Figure S2. SEM images of the BPDI (a), BB (b), and BB/BPDI (c); TEM images of the BPDI (d), BB (e), and BB/BPDI (f).

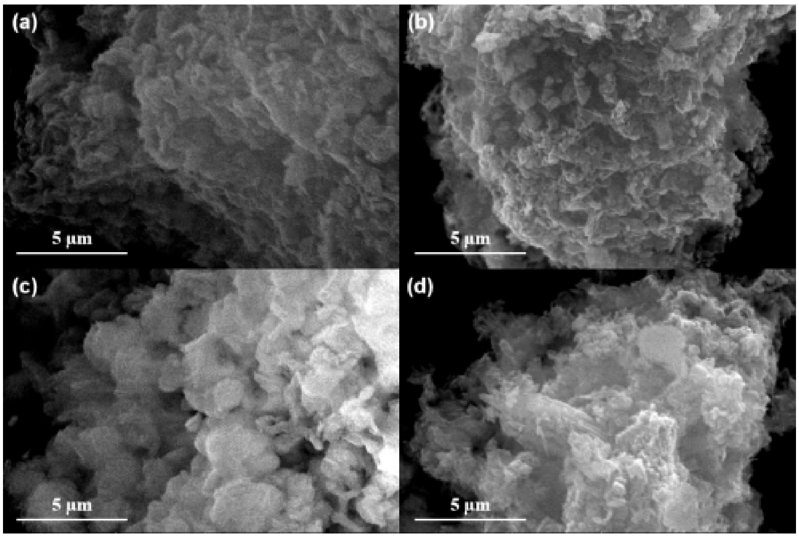

Figure S3. SEM images of the BEDOT (a), BEDOT /BPDI (b), BSO (c), BSO /BPDI(d).

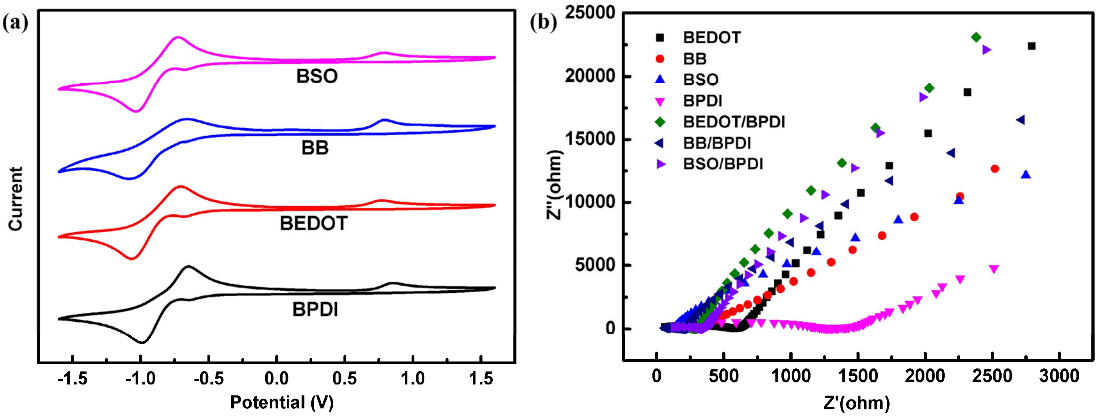

Figure S4. (a) CV curve of BSO, BB, BEDOT, and BPDI; (b) The electrochemical impedance spectra of BSO, BB, BEDOT, BPDI, BSO/BPDI, BB/BPDI, and BEDOT/BPDI.
